# The role of neurohormonal blockers in the primary prevention of acute-, early-, and late-onset anthracycline-induced cardiotoxicity

**DOI:** 10.1186/s43044-020-00090-0

**Published:** 2020-09-11

**Authors:** Ahmed Ayuna, Nik Abidin

**Affiliations:** Salford Royal NHS Foundation Trust, Manchester, UK

**Keywords:** Anthracycline-induced cardiotoxicity, Primary prevention, Neurohormonal blockers ACEI, Beta-blockers, ARB, MRA

## Abstract

**Background:**

Anthracycline-induced cardiotoxicity has been classified based on its onset into acute, early, and late. It may have a significant burden on the quality and quantity of life of those exposed to this class of medication. Currently, there are several ongoing debates on the role of different measures in the primary prevention of cardiotoxicity in cancer survivors. Our article aims to focus on the role of neurohormonal blockers in the primary prevention of anthracycline-induced cardiotoxicity, whether it is acute, early, or late onset.

**Main body of the abstract:**

PubMed and Google Scholar database were searched for the relevant articles; we reviewed and appraised 15 RCTs, and we found that angiotensin-converting enzyme inhibitors (ACEI) and B-blockers were the most commonly used agents. Angiotensin II receptor blockers (ARBs) and mineralocorticoid receptor antagonists (MRAs) were used in a few other trials. The follow-up period was on the range of 1–156 weeks (mode 26 weeks). Left ventricular ejection fraction (LVEF), left ventricular diameters, and diastolic function were assessed by either echocardiogram or occasionally by cardiac magnetic resonance imaging (MRI). The occurrence of myocardial injury was assessed by troponin I. It was obvious that neurohormonal blockers reduced the occurrence of LVEF and myocardial injury in 14/15 RCTs.

**Short conclusion:**

Beta-blockers, especially carvedilol and ACEI, especially enalapril, should be considered for the primary prevention of acute- and early-onset cardiotoxicity. ARB and MRA are suitable alternatives when patients are intolerant to ACE-I and B-blockers. We recommend further studies to explore and establish the role of neurohormonal blockers in the primary prevention of the acute-, early-, and late-onset cardiotoxicity.

## Background

Anthracycline therapy has an important role in the management of different types of cancer over the last few decades [1, 2]. Unfortunately, anthracycline-induced cardiotoxicity is one of the recognized side effects; they are, however, still in use due to their high potency in destroying cancer cells [3]. Cardiotoxicity can manifest differently, including a reduction in LV ejection fraction (LVEF), an increase in LV dimensions, impairment of LV diastolic function, and biochemical, imaging, and cellular markers of myocardial injury. Based on the time of its occurrence, cardiotoxicity can be classified into 3 main groups, acute (immediately after administration of chemotherapy up to 2 weeks), early (2 weeks to 1 year), and late (> 1 year). The probability of recovery and reversibility is less for the groups with early and late onset, especially more so for the latter group [4].

The American Society of Clinical Oncology (ASCO) in 2017 recommended cardioprotective measures for patients with high cardiovascular risk before starting anthracycline therapy. It recommends the use of ACEI, B-blockers, or a combination of both in these patient groups [5]. Similarly, ESC position paper 2016 suggested ACEI, B-blockers, and ARBs as potential cardioprotective measures [6]. ESC and ASCO are in consensus that cardiotoxicity is considered present if LVEF decline > 10% points from baseline (> 50%) or global LVEF is < 50%.

In this article, we will review the evidence for the role of neurohormonal blockers (ACEI, BB, ARB, and MRA) in the primary prevention of acute, early, and late-onset anthracycline-induced cardiotoxicity (AIC) (Table 1).

**Table 1:** The evidence for the role of neurohormonal blockers (ACEI, BB, ARB, and MRA) in the primary prevention of acute, early, and late-onset anthracycline-induced cardiotoxicity (AIC)

RCTs	Sample size	Follow-up period	Neurohormonal blocker	Parameters assessed	Outcomes in reducing cardiotoxicity
Cardinale et al. [7]	114	12 months	Enalapril	LVEF, LVEDD, LVESD, troponin I	Benefit
OVERCOME trial (Bosch et al. [8])	90	6 months	Enalapril, carvedilol	LVEF, LV diastolic function	Benefit
MANTICORE 101-BREAST study (Pituskin et al. [9])	99	350 ± 18 days	Perindopril, bisoprolol	LVEF, LVEDVI	Benefit for LVEF but not benefit for LVEDVI (both interventions)
Gupta et al. [10]	84	6 months	Enalapril	LVEF, troponin, NTproBNP, creatine kinase (CK)	Benefit
Guglin et al. [11]	468	2 years	Carvedilol, lisinopril	LVEF	Benefit
Georgakopoulos et al. [12]	147	1–3 years	Metoprolol, enalapril	LVEF	No benefit
Maryam et al. [13]	91	30 days	Carvedilol	LVEF, diastolic function, troponin I	Benefit
Tashakori Beheshti et al. [14]	70	1 week	Carvedilol	LVEF, strain, strain rate	Benefit
Kalay et al. [15]	25	6 months	Carvedilol	LVEF, diastolic dysfunction	Benefit
Abuosa et al. [16]	154	6 months	Carvedilol	LVEF, diastolic dysfunction	Benefit
CECCY trial (Avila et al. [17])	200	6 months	Carvedilol	LVEF, troponin I, BNP	Possible benefit
Kaya et al. [18]	45	6 months	Nebivolol	LVEF, LVEDD, LVESD, NTproBNP	Benefit
PRADA trial Gulati et al. [19]	130	10–61 weeks	Candesartan, metoprolol	LVEF, troponin I, BNP	Benefit Candesartan. Benefit candesartan + metoprolol. No benefit metoprolol.
Cadeddu et al. [20]	49	One week after each chemo cycle	Telmisartan	LVEF, strain, and strain rate. IL-6, reactive oxygen species.	Benefit
Akpek et al. [21]	83	24.0 ± 3.9 weeks for intervention and 24.3 ± 2.9 on placebo	Spironolactone	LVEF, LVEDD, LVESD, LV diastolic function, troponin I, NTproBNP	Benefit

It demonstrates sample size, follow-up period, type of neurohormonal blockers, parameter assessed, and the outcomes from 15 randomized controlled trials

## Main text

### Methods

PubMed database and Google Scholar were searched using the following terms: Anthracycline-induced cardiotoxicity, primary prevention of anthracycline-induced cardiotoxicity, ACEI, ARB, and Beta-blockers. Literature research was carried out from 15 January 2020 to 01 June 2020.

### Results

#### The role of ACEI

Based on experimental animal models, researchers have shown that ACEIs provide protection for cardiac function decline due to its inhibition of the renin-angiotensin-aldosterone system (RAAS), which plays a crucial role in the development of anthracycline-induced cardiac toxicity [22, 23].

Cardinale et al. [24] conducted a randomized controlled, open-labelled trial; 114 cancer patients (72 females, mean age 45 ± 12 years) were enrolled (78% previous chemotherapy, 5% hypertensive, 2% diabetic, and 4% hypercholesterolemia). All the participants had raised troponin I (TnI) enzyme levels after receiving anthracyclines. This group of individuals was thought to have a poor prognosis, especially if associated with low LVEF [7, 25]. Participants were randomized to receive either enalapril (5–20 mg per day) or no treatment over 12 months. LVEF was reassessed by transthoracic echocardiogram at baseline and the end of 12 follow-ups [24]. The primary endpoint of the study was the presence of cardiac dysfunction in the form of absolute reduction of LVEF by > 10% compared with baseline. Forty-three percent of the control group reached the primary endpoint, but none of the enalapril group did (*p* < 0.001). Additionally, there was an increase in LVEDV and LVESV in the control group only. Thirty-one cardiac events happened during the 12 months of follow-up. Only one event in the enalapril group occurred in the form of arrhythmia requiring treatment. Cardiac death (*n* = 2, *p* = 0.49), acute pulmonary oedema (*n* = 4, *p* = 0.07), HF (*n* = 14, *p* < 0.001), and arrhythmia requiring treatment (*n* = 10, *p* = 0.01) were seen in the control group [24]. The results were favourable in the use of ACEIs for primary prevention in acute and early cardiac dysfunction in the presence of anthracycline-induced myocardial injury.

In 2018, Gupta and colleagues [10] studied 84 patients with leukaemia and lymphoma (41 and 43 patients, respectively). In contrast to the earlier study, this was a randomized, double-blind, controlled trial. All the patients received doxorubicin/daunorubicin at a cumulative dose of ≥ 200 mg/m^2^. Those patients were equally randomized to receive either enalapril (*n* = 44) or placebo (*n* = 40) for 6 months. The primary endpoint was a decline of LVEF ≥ 20% from baseline (> 50% global LVEF). Troponin I, creatine kinase, and NTproBNP biomarkers were performed at baseline and 6 months later [10]. LVEF reduction was more or less the same in both groups (62.25 ± 5.49) versus ACEI (56.15 ± 4.79) (*p* < 0.001). However, a decrease of ≥ 20% of LVEF was seen in 3 patients in the placebo arm, but none in the ACE-I arm. Interestingly, NTproBNP levels were significantly raised in the placebo group after 6 months (49.60 ± 35.97) compared with the intervention group (98.60 ± 54.24) (*p* < 0.001), and cTnI (0.01 ± 0.00) in placebo vs (0.011 ± 0.003) in the intervention group (*p* = 0.0035), but not CK (1.08 ± 0.18) in the former vs (1.21 ± 0.44) in the latter (*p* = 0.079). Furthermore, none developed heart failure or arrhythmia [10]. Despite the small sample size, the results of this study were consistent with other trials which showed the beneficial role of ACEI in reducing the risk of acute and early cardiotoxicity.

#### The role of the combination of ACEI and beta-blocker

OVERCOME trial by Bosch et al. [8] evaluated the combination of enalapril and carvedilol vs placebo in the primary prevention of cardiotoxicity (1:1), *N* = 90 (mean age for participants was 50 ± 13 years) of patients with haematological malignancies receiving anthracycline therapies. All the patients included had preserved LVEF (≥ 50%) at baseline (57% males, 16% hypertensive, 11% hypercholesterolemia, 3% diabetes mellitus, and 19% smokers). At least 24 h before the first cycle of chemotherapy, enalapril and carvedilol were started simultaneously. The maximum dose of enalapril was 5 mg BD or 10 mg BD, and for carvedilol, it was 25 mg BD. Doses were adjusted according to blood pressure response. Patients were monitored for any signs of heart failure, bradyarrhythmia, hypotension, and significant deterioration in kidney function (creatinine raise > 25% of baseline reading) [8]. The primary endpoint was a change in LVEF > 10% from baseline. LVEF was checked for all patients after 6 months by echocardiography as well as cardiac MRI. Patients on enalapril and carvedilol demonstrate a non-significant trend of a lower incidence of LVEF reduction (> 10% decrease of LVEF from baseline 9.5% for intervention arm vs 19% for the placebo, *p* = 0.22) [8]. Almost all of the participants tolerated the medications very well. In summary, the combination of carvedilol and enalapril may reduce the incidence of LV systolic dysfunction in acute and early AIC.

#### ACEI vs beta-blockers

MANTICORE 101-BREAST study by Pituskin et al. [9] is a double-blind, randomized controlled trial, and 99 participants (mean age 52 ± 10) were randomized to perindopril, bisoprolol, or matching placebo (1:1:1). The primary endpoint was the decline in LVEF > 10% from baseline and < 50%. All participants received trastuzumab as an adjunctive therapy; however, one-quarter of them received anthracycline.

Cardiac MRI was used to assess LVEF. Patients were observed for 350 ± 18 days. Perindopril 2 mg, bisoprolol 2.5 mg daily, or one dose of level 1 placebo was given to the patients. Medications were up-titrated to 8 mg perindopril, 10 mg bisoprolol, or level 3 placebo tablets within 3 weeks. The maximum dose was generally well-tolerated in the perindopril, bisoprolol, and placebo arms, 75%, 65%, and 90%, respectively [9]. Patients who received placebo were a threefold higher risk of developing LVEF decline (*n* = 1 perindopril, *n* = 1 bisoprolol, *n* = 6 placebo; *p* = 0.03). Interestingly, perindopril or bisoprolol did not reduce or minimize LV remodelling for those patients who had received trastuzumab only or trastuzumab and anthracycline therapy.

A more recent trial included 468 women with non-metastatic breast cancer (age 51 ± 10.7 years) [11]. It was a randomized, multicentre, double-blind, and placebo-controlled trial. The primary endpoint was > 10% decline of LVEF and LVEF < 50% as well as treatment interruptions of trastuzumab (treated for 12 months) due to the development of cardiotoxicity. The follow-up period was for 2 years. Patients were stratified by anthracycline use and then randomized to take lisinopril, carvedilol, or placebo. Cardiotoxicity events were higher in the placebo group (47%) compared with 37% and 31% in lisinopril and carvedilol groups, respectively. Additionally, cardiotoxicity-free survival was longer on both carvedilol (HR 0.49; 95% CI 0.27–0.89; *p* = 0.009) and lisinopril (HR 0.53; 95% CI 0.30–0.94; *p* = 0.015) than on placebo. Patients on active therapy with either ACEI or B-blockers, on both the anthracycline arm and whole cohort, had experienced less interruption in their trastuzumab therapy secondary to cardiotoxicity [11]. This trial approved potential benefits for ACEI and B-blocker on acute, early, and possibly late cardiotoxicity.

Georgakopoulos et al. [12] enrolled 147 patients with lymphoma who were receiving anthracycline chemotherapy. Patients were randomized for three main groups: metoprolol, enalapril, and placebo. Baseline LVEF was 67.7% for the metoprolol group, 65.2% for the enalapril group, and 67.6% for the control group. After 1–3 years of follow-up, the authors reported that HF was less frequent under concomitant treatment than those without treatment (*n* = 1 metoprolol, *n* = 2 enalapril, *n* = 3 placebo; *X*^2^ = 1.178; *p* = 0.555) [12]. The reasons could be lower cumulative anthracycline dose in this trial, study design, and different types of malignancy.

#### The role of beta-blockers

Maryam et al. [13] conducted a single-blind, placebo-controlled study, which enrolled 91 females with breast cancer who were to receive anthracycline chemotherapy. Patients were randomly assigned to carvedilol (*n* = 46) and placebo (*n* = 45). Echocardiogram was performed before and 6 months after the chemotherapy. Both LVEF and diastolic function were assessed. TnI was checked before and 30 days after the initiation of chemotherapy. LVEF was significantly reduced in the placebo group compared with the carvedilol group (*p* < 0.001). Moreover, TnI was significantly higher in the control group compared with the intervention arm (*p* = 0.036) [13].

Another double-blind, randomized controlled study in 2016 enrolled 70 patients with breast cancers who were candidates to receive doxorubicin. Thirty patients were randomized for carvedilol (6.25 mg daily) vs placebo. Interestingly, patients were evaluated 1 week before chemotherapy and 1 week after chemotherapy for LVEF, strain, and strain rate via echocardiogram. There was a significant reduction in all targeted parameters in the placebo group compared with the intervention group (*p* < 0.001) [14]. Although the results from this trial were very encouraging, the follow-up was very short and not even enough to monitor for the development of early cardiac toxicity. So this study suggested that carvedilol might be useful in providing primary prevention of acute cardiac toxicity even at a low dose.

One of the earliest trials on carvedilol was a smaller study from 2006 with a sample size of 25 participants. Patients were randomly assigned to either carvedilol or placebo group. Those on the carvedilol arm received 12.5 mg once daily for 6 months. All patients received anthracycline chemotherapy. The mean LVEF in the carvedilol group was almost unchanged at baseline and after the chemotherapy (70.5 vs 69.7 respectively, *p* = 0.3) [15]. In comparison with the control group, there was a significant reduction in the mean EF, i.e. > 10% decline from baseline (68.9 vs 52.3; *p* < 0.001). A significant reduction in LV systolic and diastolic diameter and diastolic function was noted on the control group compared with the intervention group. An obvious limitation of this study is the small sample size.

Another interesting trial investigated the role of b-blockers in preventing AIC [16]. It was a prospective, randomized, double-blind, controlled study involving 154 patients treated with doxorubicin. Participants were randomized to either carvedilol 6.25 mg/day, 12.5 mg/day, 25 mg/day, or placebo (1:1:1:1). By the end of 6 months of follow-up, only one patient of the 116 who received carvedilol developed an LVEF decline of > 10% from baseline compared with three from the placebo group. Compared with baseline echocardiogram, there were no significant changes among the 3 different groups of carvedilol (placebo: baseline EF 62.0 ± 4.6, 6 months 58.2 ± 6.6, *p* = 0.002; 6.25 mg: baseline 61.4 ± 3.9, 6 months 60.0 ± 2.9, *p* = 0.059; 12.5 mg: baseline 60.0 ± 4.1, 6 months 58.2 ± 6.6, *p* = 0.100; 25 mg: baseline 60.4 ± 4.2, 6 months 59.2 ± 2.8, *p* = 0.07) [16]. Carvedilol may have had a preventative effect on LVEF as more patients developed LV systolic dysfunction on the placebo arm. However, there were no significant changes in diastolic function, clinical overt heart failure, or death between the two arms of the study.

CECCY trial was a prospective, double-blind, randomized (1:1), placebo-controlled trial [17], *N* = 200 (mean age 50.80 ± 10.10, *p* = 0.14) with breast cancer. All of them were planned to receive doxorubicin with a total cumulative dose of 240 mg/m^2^. All of them had an LVEF > 50% before enrolment, and the primary endpoint was to prevent ≥ 10% reduction of LVEF at 6 months. The secondary outcome was to assess the effect of carvedilol on TnI, BNP, and LV diastolic function. Medications were initiated on the first day of chemotherapy, then up-titrated at 3-week intervals to a maximum dose of 25 mg BD of carvedilol. All the patients had their echocardiogram, TnI, and BNP checked at baseline and a median of 19 days after each cycle of chemotherapy. The primary endpoint was similar in each group, so carvedilol did not result in significant changes in LV function in 6 months [17]. Nevertheless, TnI significantly raised in the placebo group compared with carvedilol, suggesting that the latter has a protective effect from myocardial injury.

One randomized controlled trial looked at the role of cardioselective B-blocker (nebivolol) in the primary prevention of AIC [18]. It was a prospective, double-blind study with 45 patients who were randomly assigned to receive nebivolol 5 mg daily (*n* = 27) or placebo (*n* = 18). Both echocardiogram and NTproBNP level were checked for all participants at baseline and 6 months of chemotherapy. By the end of 6 months, LVEF, LVEDD, and LVESD were all preserved and unchanged in the nebivolol group (LVESD 30.4 ± 3.5 to 31.0 ± 3.6 mm, *p* = 0.20; LVEDD 47.0 ± 4.4 to 47.1 ± 4.0 mm, *p* = 0.93), whereas in the placebo group, LVEF declined (57.5 ± 5.6% vs 63.8 ± 3.9%, *p* = 0.01) and both LVESD and LVEDD increased in the placebo group (LVESD 29.7 ± 3.4 to 33.4 ± 4.5 mm; LVEDD 47.2 ± 3.8 to 52.0 ± 4.6 mm, *p* = 0.01 for both). NTproBNP increased in the placebo group (144 ± 66 to 204 ± 73 pmol/l, *p* = 0.01) but remained static in the nebivolol group (147 ± 57 to 152 ± 69 pmol/l, *p* = 0.77) [18]. Thus, nebivolol has a protective role against acute and potentially early cardiotoxicity in cancer survivors who received anthracyclines.

#### ARB vs beta-blockers

In the PRADA trial, 130 patients who were recruited with early breast cancer (mean age 50 ± 10.2 years) were scheduled to receive epirubicin [19, 26]. It was a 2 × 2 factorial, single-centre, randomized, placebo-controlled, double-blind trial. The primary endpoint was to assess for any change in LVEF from baseline by cardiac MR. A 5% change was considered clinically important and significant. Participants were randomly assigned to one of the following interventions (1:1:1:1): candesartan cilexetil 32 mg/day and metoprolol succinate 100 mg/day (*n* = 32), candesartan cilexetil 32 mg/day and placebo once per day (*n* = 33), metoprolol succinate 100 mg/day and placebo once per day (*n* = 32), and placebo once per day and placebo once per day (*n* = 33). Interventions were initiated before the chemotherapy, and dose up-titration was considered every 3 days. The starting dose for candesartan was 8 mg and a maximum of 32 mg per day; for metoprolol, it was 50 mg and a maximum of 100 mg per day. Both TnI and BNP were measured at baseline and after chemotherapy.

After 10–61 weeks of follow-up, changes in LVEF in different groups compared with placebo-placebo arm [− 2.8 (95% CI − 4.3, − 1.3)] showed that reduction in LVEF was significantly less in the candesartan-placebo arm compared with the placebo-placebo arm [− 0.9 (95% CI − 2.3, 0.4); *p* = 0.025]. There was a less significant reduction in the candesartan-metoprolol arm than in the placebo-placebo arm [− 0.6 (95% CI − 2.1, 0.8); *p* = 0.075]. Moreover, there was no significant difference noted between the metoprolol-placebo arm and placebo-placebo arm [− 2.5 (95% CI − 3.9, − 1.1); *p* = 0.71] [19, 26]. Interestingly, BNP increased in metoprolol arms but not in the candesartan arms in the absence of development of symptomatic LV dysfunction; this is likely due to its relatively high intra- and inter-individual variability [27]. These findings suggest that attenuation of myocardial injury may not be reflected on the changes in LV systolic function.

#### The role of ARB

The role of telmisartan was assessed in an RCT of 49 participants [20]. One week before the chemotherapy, 25 were randomized to receive 40 mg/day of telmisartan and 24 received placebo. Echocardiogram, as well as serum samples, including reactive oxygen species and IL-6, was checked before and 7 days after every new dose of epirubicin (100 mg/m^2^). LV strain and strain rate were assess using standard echo techniques [20]. Interestingly, impairment in the strain rate reached its peak with a cumulative dose of 200 mg/m^2^ without any differences between the two arms of the study, the intervention and placebo (1.41 ± 0.31 vs 1.59 ± 0.36/s). With increasing cumulative dose, strain rate normalized only on the intervention arm at the dose of 300 mg/m^2^ and 400 mg/m^2^ (1.69 ± 0.0.42 vs 1.34 ± 0.18/s; *p* < 0.001 and 1.74 ± 0.27 vs 1.38 ± 0.24/s, *p* < 0.001) respectively. A significant increase in reactive oxygen species and IL-6 was observed in the placebo arm without any significant change on the telmisartan arm [20]. These findings indicate that ARB has an effective role in antagonizing the inflammatory effects of chemotherapy as well as its potential effect on reversing early myocardial impairment.

#### The role of MRA

Akpek and colleagues enrolled 83 female patients with breast cancer and underwent anthracycline therapy [21]. It was a prospective, randomized, double-blind, and placebo-controlled study. Participants were randomized to spironolactone 25 mg OD (*n* = 43, mean age 50 ± 11 years) and placebo group (*n* = 40, mean age 51 ± 10 years). A fixed-dose of spironolactone was given, and medications were started 1 week before starting chemotherapy. The total period of spironolactone treatment was 24.0 ± 2.9 weeks and 24.3 ± 2.9 on the placebo arm (*p* = 0.642). 2D echocardiogram was performed at baseline and repeated 3 weeks after the last chemotherapy cycle to assess LVEF, LVEDD, LVESD, and LV diastolic function. Additionally, TnI and NTproBNP were checked in both groups. There was a significant decline in LVEF in the control group compared with the spironolactone group (67.7 ± 6.3 to 53.6 ± 6.8; *p* < 0.001 vs 67.7 ± 6.1 to 65.7 ± 7.4; *p* = 0.094). Besides, participants on the spironolactone arm had preserved LV diastolic parameters (*p* = 0.096), whereas significantly deteriorated in the control group (*p* < 0.001). The TnI level was significantly higher in the control group compared with the intervention group (*p* = 0.006). Serum NTproBNP level increased in both groups without significant differences between the two groups [71 (48–125 pg/ml) to 85 (51–100 pg/ml); *p* = 0.549 vs 70 (56–72 pg/ml) to 100 (89–138 pg/ml); *p* = 0.089] [21]. In summary, spironolactone is an effective medication in the primary prevention of acute- and early-onset AIC. It provided potential protection for LV systolic and diastolic function as well as myocardial injury.

#### Discussion

The data from our research suggested that neurohormonal blockers reduced the reduction in the LVEF and myocardial injury in 14/15 RCTs. The primary endpoint was set almost similar in all the studied RCTs; however, there were slight differences in the target decline in LVEF, almost all of the trials, the target was ≥ 10% unit decline from baseline LVEF and the baseline LVEF should be > 50% apart from two trials: MANTICORE 101-BREAST and Gupta et al. (2018) which was > 5% and > 20% decline from baseline, respectively. Total participants (*N*) from all RCTs were 1849; they had either haematological or non-haematological malignancies. All of the participants received anthracyclines, apart from a small number of patients (*n* = 74) who received trastuzumab only in the MANTICORE 101-BREAST study. The rest of the participants from this study (*n* = 25) received both anthracycline and trastuzumab, and the results were similar in each group and parallel to the other trials suggesting a benefit from both b-blockers and ACEI.

The sample size was generally small in all the trials. Besides, most of the RCTs carefully selected their participants with minimal cardiovascular risk and comorbidities, and they were at their middle age. Despite that, the neurohormonal blockers’ safety and efficacy were shown suggesting that they might be even more effective in those who have underlying comorbidities like hypertension, coronary artery disease, diabetes mellitus, and chronic kidney disease.

Most of the patients involved in these studies were of middle age rather than elderly patients who are more liable to develop cancer. This can be considered as a limitation for these trials.

Almost all the participants tolerated well these medications, with no issues of poor adherence. Interestingly, the use of different doses of carvedilol was safe and effective in reducing cardiotoxicity. Furthermore, a combination of two drugs like carvedilol and enalapril was observed to be an effective measure to minimize cardiotoxicity.

The imaging modality used in almost all the RCTs was the 2D echocardiogram. Cardiac MRI is more sensitive and accurate in the assessment of LV function. However, due to its availability and cost-effectiveness, the authors’ opinion is to use echocardiogram as the first-line imaging modality. In those with poor echocardiographic window, a contrast echocardiogram can be used to enhance LV endocardial borders and accurate measurements of LVEF using Simpson’s biplane method. If still difficult, cardiac MRI could be considered if available, no contraindications like claustrophobia, or significant renal impairment due to risk of gadolinium-induced nephrotoxicity. The role of the neurohormonal blockers in preventing LV diastolic function is still controversial, especially the B-blocker, and further research with a large sample size is recommended.

Furthermore, all the trials focused on preventing acute and early cardiotoxicity. Follow-up for all RCTs was 1–156 weeks (mode 26 weeks). Evidence is less available about the prevention of late-onset cardiotoxicity, i.e. > 1 year after chemotherapy.

## Conclusions

There are now several small trials with positive outcomes on the use of neurohormonal blockers in primary prevention of anthracycline-induced cardiotoxicity. Based on the currently available evidence, it is prudent to consider those patients, especially with high-risk CV factors for B-blockers, ACEI, or a combination of both. If intolerant to these medications, other options such as ARB and MRA could be considered. These measures may potentially reduce the risk of acute and early onset cardiotoxicity. Further research is required to study the effects of the medications in patients with anthracycline-induced acute-, early-, and late-onset cardiotoxicity.

## Data Availability

Not applicable

## Abbreviations

2D: Two dimensional
ACEI: Angiotensin-converting enzyme inhibitor
AIC: Anthracycline-induced cardiotoxicity
ARB: Angiotensin receptor blocker
ASCO: American Society of Clinical Oncology
B-blocker: Beta-blocker
BD: Twice a day
BNP: Brain-type natriuretic peptide
CK: Creatine kinase
Cardiac MRI: Cardiac magnetic resonance imaging
ESC: European Society of Cardiology
HF: Heart failure
IL-6: Interleukin-6
LV: Left ventricle
LVEDD: Left ventricular end-diastolic diameter
LVEDV: Left ventricular end-diastolic volume
LVEF: Left ventricular ejection fraction
LVESD: Left ventricular end-systolic diameter
LVESV: Left ventricular end-systolic volume
MRA: Mineralocorticoid receptor antagonist
RAAS: Renin-angiotensin-aldosterone system
RCT: Randomized controlled trial
TnI: Troponin I
